# Assessing phylogenetic confidence at pandemic scales

**DOI:** 10.1038/s41586-025-09567-x

**Published:** 2025-11-05

**Authors:** Nicola De Maio, Nhan Ly-Trong, Samuel Martin, Bui Quang Minh, Nick Goldman

**Affiliations:** 1 European Bioinformatics Institute, European Molecular Biology Laboratory, Hinxton, UK; 2 School of Computing, College of Systems and Society, Australian National University, Canberra, Australian Capital Territory Australia

**Keywords:** Molecular evolution, Phylogenetics, Phylogeny, Classification and taxonomy, Statistical methods

## Abstract

Phylogenetics has a central role in evolutionary biology and genomic epidemiology^1^. Assessing phylogenetic confidence and reliability is therefore crucial and the methods that do this, such as those derived from Felsenstein’s bootstrap^2^, are among the most widely used in modern science. However, these methods require enormous computational capacity, and are unsuitable for large datasets. Furthermore, most of these methods emerge from a focus on the membership of clades (groupings of taxa), which makes their results difficult to interpret in the context of genomic epidemiology. Here we propose subtree pruning and regrafting-based tree assessment (SPRTA), an efficient and interpretable approach to assess confidence in phylogenetic trees. SPRTA shifts the paradigm of phylogenetic support measurement from evaluating the confidence in clades to evolution histories and phylogenetic placement—for example, assessing whether a lineage evolved from another considered lineage, which is particularly valuable in genomic epidemiology. We use SPRTA to investigate a global public SARS-CoV-2 phylogenetic tree relating more than two million genomes, highlighting plausible alternative evolutionary origins of many SARS-CoV-2 variants, assessing reliability in the Pango outbreak lineage classification system^3^, and demonstrating the effect of phylogenetic uncertainty on inferred mutation rates. Our results show that SPRTA enables pandemic-scale and detailed probabilistic assessment of transmission and mutational histories. Our method introduces a new approach to assessing phylogenetic confidence, enhancing the interpretability of pandemic-scale phylogenetic analyses and improving our ability to prepare for and respond to future pandemics.

## Abstract (web summary)

A method using subtree pruning and regrafting-based tree assessment (SPRTA), which considers evolutionary relationships between lineages, enhances interpretability of phylogenetic analyses such as those of SARS-CoV-2 variants.

## Main

Phylogenetics is central to evolutionary biology^1^. Phylogenetic trees are graphs representing evolutionary histories and ancestry, and are typically inferred from the genomic data. In genomic epidemiology, for example, DNA sequences of the same pathogen are collected from different hosts (for example, SARS-CoV-2 genomes collected from different patients) and compared with one another. Phylogenetic trees inferred from these genomes can reveal the emergence of drug resistance and new variants of concern, transmission between individuals and countries, and many other details of the evolution and spread of the pathogen^4^.

Most phylogenetic methods that are scalable to large datasets—such as maximum-likelihood, parsimony-based and heuristic approaches—typically estimate a single phylogenetic tree without intrinsically assessing the reliability or uncertainty of inferences. This issue is typically addressed with additional methods such as Felsenstein’s bootstrap^2^. For a given dataset, Felsenstein’s bootstrap typically creates 100 or 1,000 replicates by randomly resampling the genetic data with replacement. Phylogenetic inference is performed on each to estimate replicate trees, and the support score of a clade (the set of taxa inferred to be all the descendants of one ancestor in the tree) is defined as the proportion of replicate trees containing that clade. This is also considered to be the support of the phylogenetic branch that separates the clade from all other taxa in the tree.

Felsenstein’s bootstrap has been developed in the context of inter-species evolutionary biology. Consequently, it has a number of drawbacks when applied to genomic epidemiological datasets of closely related genetic sequences. First, repeatedly performing phylogenetic estimation on all replicate datasets can be excessively computationally demanding. Although Felsenstein’s bootstrap approximations^5–7^ are more efficient, these are still not feasibly applicable to pandemic-scale phylogenetic analyses involving millions of genomes^8,9^.

Second, even a small number of ‘rogue taxa’—that is, sequences whose placement in the inferred phylogenetic tree is highly uncertain (such as incomplete sequences or recombinants)—can substantially lower the Felsenstein’s bootstrap support of many internal branches throughout phylogenetic trees^10^. Third, Felsenstein’s bootstrap does not measure posterior probability, but rather measures repeatability^11^. In genomic epidemiology, a single mutation is typically sufficient to define a clade with negligible uncertainty. However, in this scenario, Felsenstein’s bootstrap usually requires three mutations supporting any one clade to be able to assign 95% support to it, making it excessively conservative^2,12^.

Finally, Felsenstein’s bootstrap and most other phylogenetic branch support measures have a ‘topological’ focus, in that they aim at assessing the reliability of the inferred tree topology via its constituent clades. Although clades are important in taxonomy, they are not as relevant in genomic epidemiology, where the focus is typically on mutation and transmission histories and lineage assignment^3,13^.

Existing local branch support measures^14–17^ are considerably more computationally efficient than Felsenstein’s bootstrap, but also rely on a topological interpretation. They usually compare the likelihood of the inferred phylogenetic tree against the likelihoods of similar alternative trees (Methods, ‘SPRTA and aBayes’). Local branch support measures are particularly appealing because of their computational efficiency and their robustness to rogue taxa.

Here, drawing on concepts from local branch support measures and the approximate Bayes approach (aBayes)^15^, we present a new measure of branch support that is robust to rogue taxa and can scale to pandemic-scale trees. Subtree pruning and regrafting-based tree assessment (SPRTA) shifts the focus from a topological point of view to one centred around the evolutionary origin of lineages (‘mutational’ or ‘placement’ focus).

## Principles of SPRTA

**Fig. 1:**
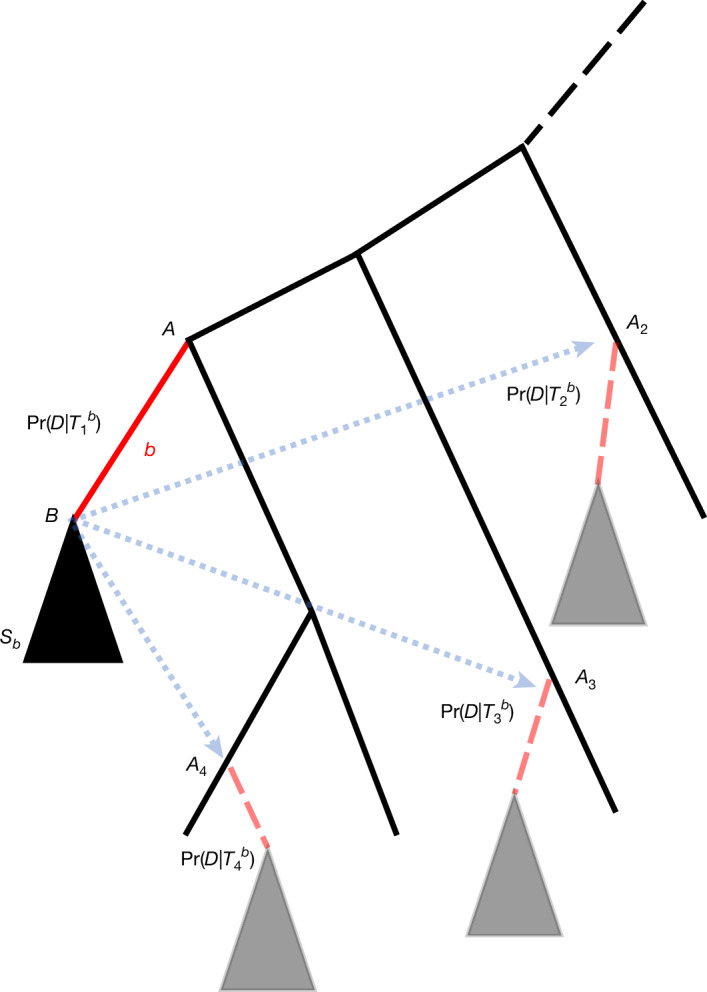
Graphical representation of SPRTA branch support measurement. Here we represent SPRTA branch support calculation for an example tree branch *b* (highlighted as a solid red line). The subtree *S*_*b*_ below *b* is represented with a black triangle and is not affected by any of the SPR moves considered to evaluate the support of *b*. *A* and *B* represent the ancestors of *b* and *S*_*b*_, respectively. Solid black lines represent the branches of *T*\*S*_*b*_, which are assessed as alternative placements for *S*_*b*_, and the dashed black line represents the rest of *T* (which is an arbitrarily large tree), not shown here. Some possible SPR moves are highlighted with dotted blue arrows, and cause hypothetical new branches (shown as shaded red dashed lines), leading to copies of *S*_*b*_ (grey triangles) descending from nodes *A*_2_–*A*_4_. The relative likelihood of the original tree is $\Pr (D| {T}_{1}^{b})$ while the likelihoods of alternative topologies are represented by $\Pr (D| {T}_{2}^{b})$, $\Pr (D| {T}_{3}^{b})$ and $\Pr (D| {T}_{4}^{b})$. In this case, the support for branch *b* will be $\Pr (D| {T}_{1}^{b})/{\sum }_{i=1}^{4}\Pr (D| {T}_{i}^{b})$. In practice, a large number *I*_*b*_ of alternative topologies might be considered for each branch *b* (Methods, ‘SPRTA and aBayes’).

As typical in molecular phylogenetics, we assume that genetic data are represented as a multiple sequence alignment *D*: a matrix in which each row corresponds to the genetic sequence of a considered taxon (in genomic epidemiology this is often the genome sequence of one of the considered samples, aligned to the reference genome) and each column contains all homologous nucleotides (those descending from the same ancestral nucleotide). We also assume we have a rooted phylogenetic tree *T* (Methods, ‘SPRTA and aBayes’) inferred from *D*. Our aim is to assign confidence scores to branches *b* of *T* (see Fig. 1). Most branch support methods assign scores that represent the confidence that the sequences contained in subtree *S*_*b*_ (black triangle in Fig. 1), containing all descendants of *b*, indeed form a clade within *T*—we refer to this as the topological focus. Instead, we want to assess the probability that *b* correctly represents the evolutionary origin of *S*_*b*_—we call this a mutational focus. To clarify this, we describe branch *b* as having immediate ancestor *A* and descendant *B* (the root of subtree *S*_*b*_), and dividing *T* into *S*_*b*_ and its complement *T*\*S*_*b*_ (Fig. 1). *A* and *B* might correspond to individual genomes if, as typical in genomic epidemiology, uncertainty regarding ancestral genomes is low; otherwise, they might represent sets of probable genomes. Our aim is to efficiently approximate the probability that *B* evolved from *A* through mutations along branch *b* (that is, *B* and *S*_*b*_ are ‘placed’ at *A*), as opposed to the alternative hypothesis that *B* descended from some other node of *T*\*S*_*b*_ (‘alternative placements’ of *S*_*b*_ (blue arrows in Fig. 1)). In other words, SPRTA(*b*) represents the confidence that branch *b* is the evolutionary origin of *B* and *S*_*b*_.

To achieve this, for each branch *b*, SPRTA considers a number *I*_*b*_ of alternative topologies ${T}_{i}^{b}$ (1 ⩽ *i* ⩽ *I*_*b*_) of *T* obtained by performing single subtree pruning and regrafting (SPR) moves—changes to *T* that relocate *S*_*b*_ as a descendant of other parts of *T*\*S*_*b*_ (Fig. 1; *I*_*b*_ is defined in Methods, ‘SPRTA and aBayes’). Each such SPR move represents a possible different origin of *B*, as a direct descendant of a node *A*_*i*_ other than *A*. For notational convenience, we assume that ${T}_{1}^{b}=T$ is the original tree topology. The likelihood $\Pr (D| {T}_{i}^{b})$ of such topologies is routinely and efficiently calculated by MAPLE^9^ (up to a constant common multiplicative factor) and we use it to define the SPRTA support score:

1 $${\rm{SPRTA}}(b)=\mbox{Pr}(b| D,T\backslash b)=\frac{\mbox{Pr}(D| T)}{{\sum }_{1\leqslant i\leqslant {I}_{b}}\mbox{Pr}(D| {T}_{i}^{b})}$$

where *T*\*b* represents the union of subtree *S*_*b*_ and its complement subtree *T*\*S*_*b*_. SPRTA is described in more detail in Methods ‘SPRTA and aBayes’.

SPRTA mutational branch support scores need to be interpreted differently from those of existing topological branch support methods. SPR moves involving branch *b* preserve the clade defined by *b* (Fig. 1), and so SPRTA scores do not represent an assessment of this clade. Rather, SPRTA scores are to be interpreted as an approximate probability that *B* evolved directly from *A*. These scores are a particularly useful assessment of tree reliability in the context of genomic epidemiology, where the placement of individual incomplete sequences (rogue taxa) can be highly uncertain. SPRTA scores are expected to be robust to rogue taxa, since their placement is expected to have negligible effect on relative likelihood scores at internal tree nodes.

SPRTA scores for the terminal branches of a tree evaluate the placement probability of individual observed sequences, and in fact correspond closely to the probabilistic support measure used by some tools that map query sequences onto a pre-estimated phylogeny^18,19^. By contrast, topological support methods cannot assess terminal branches and sequence placements.

## Computational demand

**Fig. 2:**
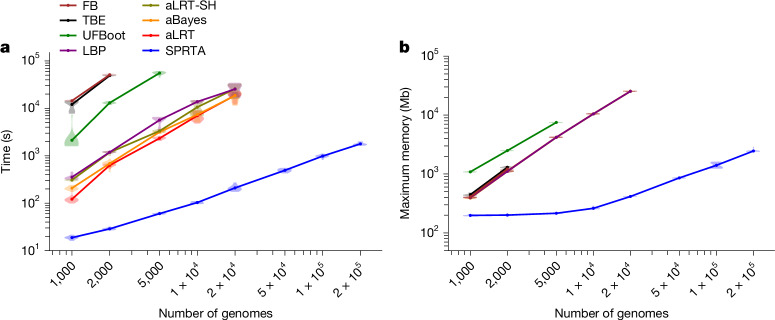
Computational demand. **a**,**b**, Time (**a**) and maximum memory usage (**b**) of different branch support methods. The *x* axis shows the number of simulated SARS-CoV-2 genomes (Methods, ‘Simulated genomes’) included in each replicate. For each dataset size considered, we ran 20 replicates. Dots show the mean across replicates and violin plots show variation between replicates. All analyses were run on one core of an Intel Xeon Gold 6252 processor at 2.10 GHz. Each method was run on the same replicates for the results in **a**,**b**. In **b**, the lines for all methods other than UFBoot and SPRTA overlap. FB, Felsenstein’s bootstrap.

**Extended Data Fig. 1:**
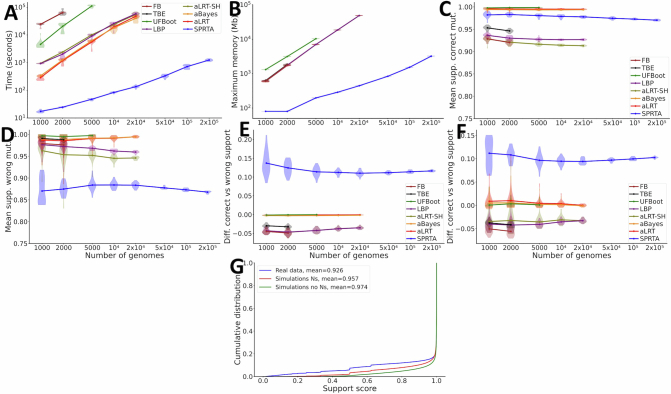
Other simulation results. **A** Time and **B** maximum RAM usage of different branch support methods on simulated incomplete genomes. In **A**, the lines for FB and TBE coincide very closely, as do those for the pairs LBP and aLRT-SH, and aBayes and aLRT. In **B**, all methods other than UFBoot and SPRTA overlap. **C** Mean support of correctly inferred mutations within each replicate in simulations with incomplete genomes. The lines for aBayes and aLRT nearly coincide, as do those for FB and aLRT-SH but with FB unable to be run for more than 2,000 genomes. **D** Mean support of wrongly inferred mutations within each replicate in simulations with incomplete genomes. **E** Difference between the mean support of correctly inferred mutation events (Fig. 3**A**), and the mean support of wrongly inferred mutation events (Fig. 3**B**) in simulations with complete genomes. **F** Same as **E** but in simulations with incomplete genomes. Other details in **A**–**F** are as in Figs. 2 and 3. **G** Cumulative distributions of SPRTA support scores of mutations in real SARS-CoV-2 data (blue, see Section 4.5.1), simulated data with incomplete sequences (red, see Section 4.5.2), and simulated data with complete sequences (green, see Section 4.5.2). For the simulated data distributions we merged the mutation support scores for all 20 replicates of the largest (200,000 sequence) simulations into a single distribution.

The SPR search required by SPRTA is typically performed as part of the phylogenetic tree search in many maximum-likelihood phylogenetic methods such as RaxML^20^ and MAPLE^9^, and so it is not expected to lead to significant additional runtime when executed in conjunction with phylogenetic inference. For comparison, we assessed the computational demand of SPRTA against the main existing measures of branch support (Felsenstein’s bootstrap^2^, local bootstrap probability (LBP)^14^, approximate likelihood ratio test (aLRT)^15^, aLRT with Shimodaira–Hasegawa test (aLRT-SH)^16^, Bayesian-like transformation of aLRT (aBayes)^17^, transfer bootstrap expectation (TBE)^10^ and ultrafast bootstrap approximation (UFBoot)^7^). SPRTA reduces runtime and memory demands by at least two orders of magnitude compared with all these methods, with the difference growing as dataset size increases (Fig. 2 and Extended Data Fig. 1a,b). Note also the premature termination of the lines in Fig. 2 and Extended Data Fig. 1 for the other methods, indicating cases where they could not be run successfully. Among these other methods, those based on Felsenstein’s bootstrap (Felsenstein’s bootstrap, UFBoot and TBE) have substantially higher computational demand than local branch support measures. This comparison shows that SPRTA can assess much larger phylogenetic trees than existing approaches.

## Accuracy

To benchmark different branch support methods, we simulated SARS-CoV-2-like genome data (Methods, ‘Simulated genomes’) for which we know the true tree and the true mutational history along the tree. Because genomes in genomic epidemiological datasets are typically very similar to each other, we usually infer ancestral genomes and mutation events conditional on an inferred phylogenetic tree *T* with negligible uncertainty. In this case the task of assessing whether *B* evolved from *A* along branch *b* is equivalent to assessing the correctness of the mutation events implied by *T* on *b*, since alternative placements of *S*_*b*_ often result in alternative mutational histories leading to *B*. Therefore, in this benchmark we consider a mutational interpretation of branch support, and interpret branch support scores as an estimate of the posterior probability of the mutation events implied by *T* on the considered branch *b*. Each individual branch of a tree inferred from simulated data is considered correct if the mutations inferred on that branch actually happened in our simulations, and so if *B* at the lower end of *b* actually evolved from *A*. An advantage of this approach is that, whereas *S*_*b*_ or the clade defined by it are often uncertain and might not exist in the real tree, *B* will typically not be affected by rogue taxa or other topological uncertainty within *S*_*b*_. More details on how we define accuracy of branch support are provided in Methods, ‘Benchmarking of SPRTA support’. This is not the typical interpretation of branch support measures other than SPRTA, and so results here are not meant as an evaluation of other branch support measures in their classical topological interpretation. Below, all branch support measures are evaluated based on their ability to assign higher support to correctly inferred mutation events, and lower support to erroneously inferred mutation events.

**Fig. 3:**
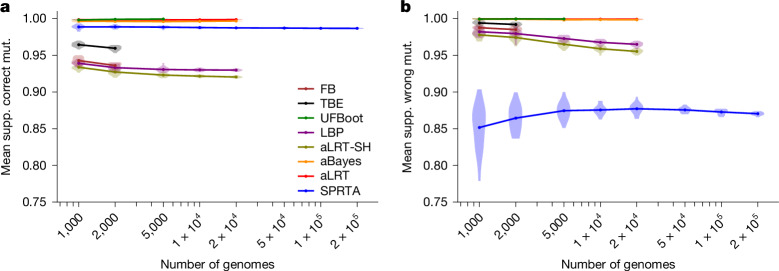
Benchmark of branch support methods. **a**, Mean support of correctly inferred mutations (supp. correct mut.) within each replicate. **b**, Mean support of wrongly inferred mutations (supp. wrong mut.) within each replicate. Note that values shown are distributions of mean support values across all mutations in a replicate simulation, and not distributions of support scores of individual branches or mutations. Other details are as in Fig. 2. Branch support scores for methods other than SPRTA were again only calculated when computationally feasible.

**Extended Data Fig. 2:**
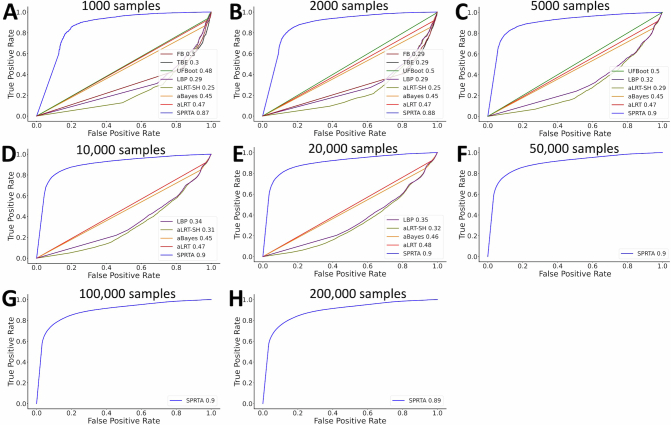
Receiver operating characteristic (ROC) curves. ROC curves for all considered methods on simulated data, from (**A**) 1,000 to (**H**) 200,000 samples. On the Y-axis we show the true positive rate of branch support scores when used to distinguish between correct and wrong mutation events. On the X-axis is the false positive rate. For each curve we combine all branches of all replicates of the same size. In the legend, next to the name of each method that could be run for this data size, we show the area under the curve (AUROC). Worse than random performance of some methods (e.g. FB and TBE) can be attributed to the fact that these support scores measure the confidence in the clade defined by the considered branch, and not in the mutation events implied by that branch (compare to Extended Data Fig. 1E).

**Extended Data Fig. 3:**
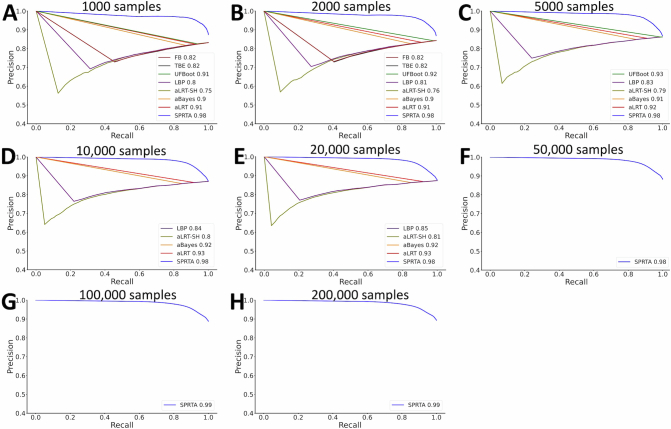
Precision-recall curves (PRC). Precision-recall curves for all considered methods on simulated data. On the Y-axis we show the precision of branch support scores when used to distinguish between correct and wrong mutation events. On the X-axis is the recall rate. Legends indicate methods that could be run for each data size and areas under their curves (AUPRC). Other details are as in Extended Data Fig. 2.

**Extended Data Fig. 4:**
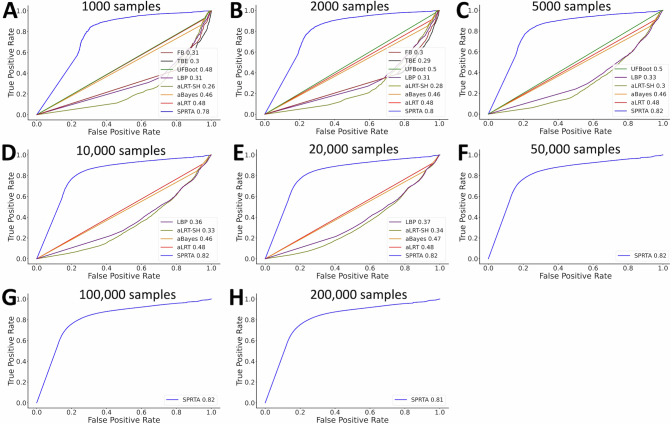
Receiver operating characteristic (ROC) curves for simulations with incomplete genomes. ROC curves for all considered methods on simulated data, from (**A**) 1,000 to (**H**) 200,000 samples, on simulations with incomplete genomes. Other details are as in Extended Data Fig. 2.

**Extended Data Fig. 5:**
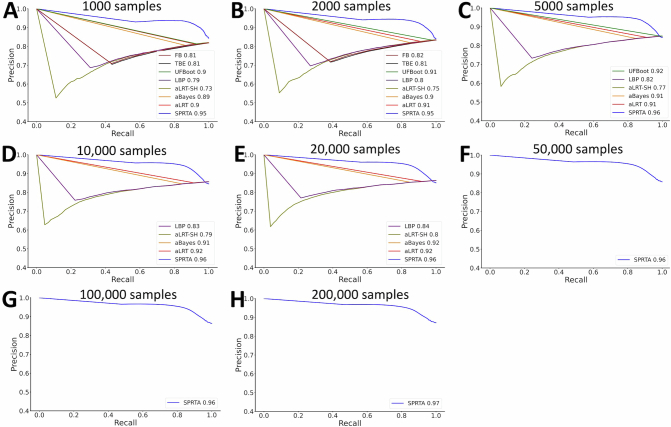
Precision-recall curves (PRC) on simulated incomplete genomes. Precision-recall curves for all considered methods on simulated incomplete genomes. Other details are as in Extended Data Fig. 3.

Generally, all methods give high support scores (>80%) to all mutations, both correctly and wrongly inferred ones (Fig. 3 and Extended Data Fig. 1c,d). This is to be expected given the low level of divergence in the considered dataset, and that these mutations have been inferred by maximum likelihood and so by definition will typically have higher likelihood than alternative mutation histories. However, SPRTA is the only method that reliably assigns higher support to correctly inferred mutation events (typically a mean of 98–99%) and lower support scores to wrongly inferred ones (typically a mean of 85–90%) (Fig. 3 and Extended Data Fig. 1e,f). Although some methods like UFBoot, aLRT, and aBayes give higher support to correctly inferred mutations than does SPRTA, they also similarly support wrongly inferred mutations, and so do not discriminate between the two. This is also reflected in higher AUROC and AUPRC values for SPRTA (Extended Data Figs. 2–5). It should be remembered, however, that topological approaches have been developed for a different interpretation of branch support scores, and so these results are not indicative of their performance for that task. What our analysis does show is that SPRTA can usefully and effectively assess the confidence in mutational histories implied by inferred phylogenetic trees.

## Uncertainty of SARS-CoV-2 evolution

We applied SPRTA to a global public dataset of 2,072,111 SARS-CoV-2 genomes (Methods, ‘Viridian genome dataset’) that is too large for the feasible application of existing topological branch support measures. Phylogenetic tree estimation with MAPLE, parallelized over 14 cores of an Intel Xeon Gold 6252 processor at 2.10 GHz, took around 10 days (ref. ^21^). Post hoc SPRTA mutational support assessment, re-performing SPR move evaluations that had already been performed during tree inference, required 7 h 27 m on a single core and maximum memory 26.93 Gb. This branch support assessment can be also performed simultaneously with tree inference, at negligible additional computational cost compared to the tree inference itself. During tree inference, however, we do not evaluate possible alternative placements of less informative genomes (a sequence is not informative at a position if it contains the ‘N’ character at that position, and a sequence *s*_1_ is less informative than *s*_2_ if it coincides with *s*_2_ except at positions where *s*_1_ is not informative), since alternative placements of these genomes do not increase the likelihood of the considered tree^21^. To evaluate alternative placements of all genomes—for example, to identify a larger number of rogue taxa in our dataset—we also performed a more in-depth and computationally demanding SPRTA assessment that did not collapse genomes less informative than other genomes in the dataset; this required 22 h 42 m on a single core and maximum memory 27.75 Gb.

### Phylogenetic uncertainty

Among the 2,072,111 genomes considered here, 636,022 are mutationally informative (the branch separating them from the tree has inferred mutations on it), but for many of these the mutational history is uncertain: 87,406 have SPRTA placement below 90% and 53,365 have SPRTA placement below 50%. From the remaining 1,436,089 non-mutationally informative genomes (those that are separated from the tree by a terminal branch of length 0 and so, conditional on the rest of the tree, are not informative of mutation events), 162,100 have SPRTA placement support below 90% and 115,358 have SPRTA placement below 50%.

Phylogenetic uncertainty also affects many internal branches of the phylogenetic tree, highlighting substantial uncertainty in the ancestral mutational history, and not only in terminal branches: from 453,976 internal branches with inferred mutations, 59,523 have SPRTA support below 90% and 29,641 have SPRTA support below 50%. In terms of inferred mutations (which are assigned the same SPRTA score as the branch they are inferred to have occurred on), out of a total of 1,827,786 there are 249,092 with SPRTA support below 90% and 126,308 with SPRTA support less than 50%.

Although in our simulations we model site-specific genome evolution and incomplete sequence data to closely match patterns observed in real SARS-CoV-2 data (Methods, 'Simulated genomes’), we still find lower SPRTA scores in real data than in simulations (Extended Data Fig. 1g). This suggests further complexities in real data such as site- and nucleotide-specific mutational patterns^22^ that are not accounted for in our models of genome evolution.

**Fig. 4:**
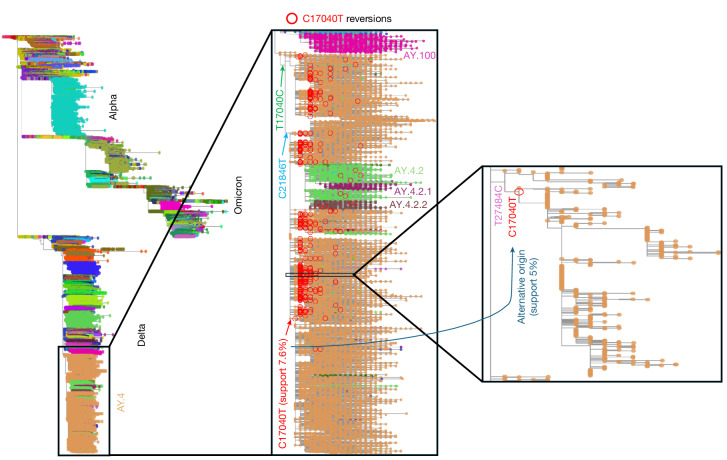
Uncertain evolutionary history of SARS-CoV-2 lineage AY.4. Left, the global SARS-CoV-2 phylogenetic tree inferred by MAPLE and visualized in Taxonium^25^. Tips are coloured according to the Pango^3^ lineage assigned by Pangolin^35^ v.4.3 (with Pangolin-data v.1.21) to the corresponding genomes. We also show the names of some of these lineages. Middle, magnified view of lineage AY.4, showing the locations of inferred T17040C (green arrow) and C21846T (blue arrow) mutations that define the inferred early evolution of the lineage. We highlight with red circles the approximately 650 C17040T reversions within AY.4. One of these, highlighted with a red arrow, is ancestral to more than 163,000 genomes and has only 7.6% SPRTA support. Right, further magnification of the location of an alternative origin of this last sub-lineage (with corresponding SPR move represented by the dark blue arrow) that has 5.0% SPRTA support and entails re-placing the aforementioned sub-lineage as a direct descendant of the phylogenetic node with genome containing the T27484C and C17040T mutations.

A prominent examples of uncertainty in the SARS-CoV-2 mutational history is the evolution of the AY.4 Delta sub-lineage, one of the most abundant SARS-CoV-2 lineages, represented here by more than 480,000 genomes. Two mutations appear to be ancestral to most AY.4 genomes: T17040C and C21846T (Fig. 4, middle). After the appearance of mutation T17040C, the reversion C17040T appears to have occurred many times: we infer around 650 reversions. Position 17040 is inferred by MAPLE to have a substitution rate 31.9 times higher than average^21^, mostly as a result of these reversions within AY.4. Although inferred reversions can be due to reference biases in consensus genome calling methods, our consensus genomes were called with Viridian^23^, which is not affected by this issue^21,23^. Furthermore, we did not observe any issues with read data or substitution distribution along the tree that would suggest the presence of recurrent sequence errors at position 17040^21^. This suggests that 17040 might be a genuinely hyper-mutable genome position in SARS-CoV-2, but only when in the mutated C nucleotide state, and not in the ancestral T. This is in line with the observation that mutation patterns in SARS-CoV-2 are highly position- and nucleotide-specific^22^.

The largest inferred sub-lineage within AY.4 descending from a C17040T reversion contains more than 163,000 genomes (Fig. 4, middle). However, the branch defining this lineage (Fig. 4, middle, red arrow) has only 7.6% SPRTA support. The reason is that, owing to the hyper-mutability of C17040, there are many alternative plausible mutational histories within AY.4. The most likely alternative origin of this sub-lineage has SPRTA support of 5.0% and involves replacing the C17040T reversion defining this lineage with a C27484T reversion within a genomic background that already contains the C17040T reversion (Fig. 4, right, dark blue arrow). Although many equally parsimonious alternative mutation histories exist, this one is inferred by SPRTA to be the most likely alternative origin of this sub-lineage because the background mutation rate from C to T is very high in SARS-CoV-2, and because position 27484 also has an inferred substitution rate 20.8 times above average^21^. These results show that the evolutionary history of lineage AY.4 is highly uncertain, and illustrate how SPRTA scores can not only highlight uncertain parts of an inferred phylogenetic tree and mutational history, but also identify and probabilistically assess alternative evolutionary origins of considered pathogen variants.

**Fig. 5:**
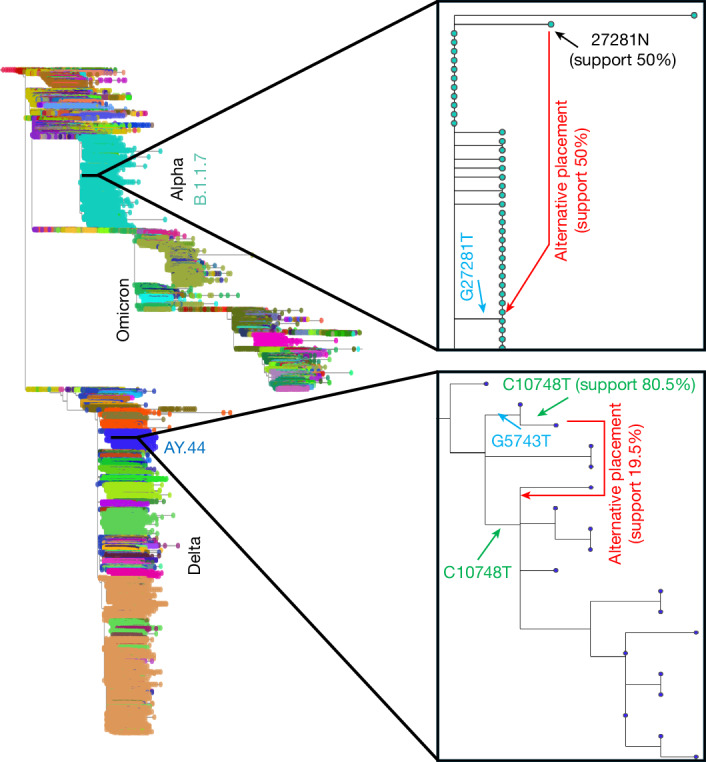
Sample placement uncertainty. Left, the global SARS-CoV-2 tree as in Fig. 4. Right, magnified view of two uncertain sample placements. Top right, within lineage Alpha, the considered sample (marked by the black arrow) has no sequence information at genome position 27281 (ambiguity character N), the same position where a G-to-T mutation occurs nearby in the phylogenetic tree (marked by the blue arrow). This sample has 50% SPRTA support both at the current placement and at the other side of G27281T (red arrow). Bottom right, within the AY.44 lineage, the placement of a sample has 80.5% SPRTA support, since the mutation C10748T implied by the current placement of the sample (top green arrow) also occurs on a nearby branch (bottom green arrow), permitting an alternative placement with 19.5% SPRTA support (red arrow). This alternative placement would require one fewer C10748T mutation on the tree but one additional G5743T mutation.

Regarding individual samples with uncertain placement, we show two examples (of many) in Fig. 5. In the top example, uncertainty is caused by the sample having an incomplete genome sequence; in the bottom example, uncertainty is caused by the existence of two mutually plausible mutational histories. This shows how SPRTA can effectively identify the uncertainty related to the placement of rogue taxa, which by definition will have many possible placements but all with similarly low SPRTA support. Other branch support methods, by contrast, cannot provide an evaluation of sample placement, as they only measure support for internal branches of the phylogenetic tree. Also, unlike for Felsenstein’s bootstrap and most other topological branch support measures, rogue taxa mostly do not affect the SPRTA support scores of ancestral nodes, as their placement typically only affects the inference of mutation events on phylogenetic branches near the placement itself.

These are only some examples of all mutations and placements in the SARS-CoV-2 tree that are substantially uncertain (126,308 out of all 1,827,786 inferred mutations have SPRTA support below 50%; see above). Our full maximum-likelihood tree annotated throughout with SPRTA support scores concisely represents the evolution of SARS-CoV-2 during the COVID-19 pandemic and presents the SPRTA probabilistic assessment of each sampled genome placement and each inferred mutation. It also offers a concise summary of plausible alternative placements and mutational histories. Such pandemic-scale probabilistic phylogenetic assessment—and in general the assessment of mutation events, placements and alternative mutational histories—is not possible with existing popular branch support methods. This annotated tree is available on Zenodo^24^ together with the considered SARS-CoV-2 genome alignment, and can be visualized easily within Taxonium^25^.

### Effect on inferred mutation rates

**Extended Data Fig. 6:**
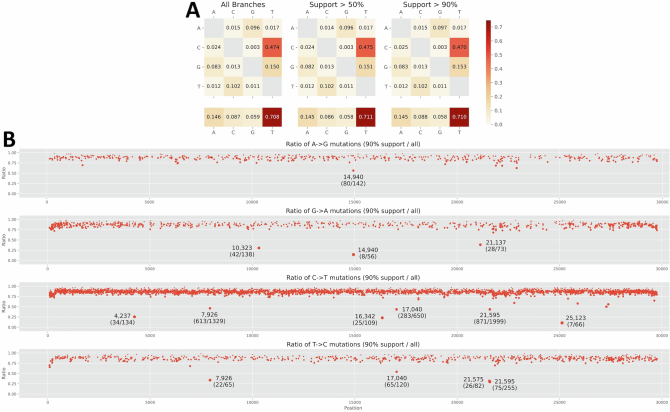
Impact of phylogenetic uncertainty on the inference of substitution rates. **A** (upper) normalized substitution counts and (lower) equilibrium base frequencies calculated from inferred rates over (left) all branches, (middle) branches with > 50% support, and (right) branches with > 90% support. Note that there are only very minor differences between the results based on mutations on branches at the three different support levels. **B** Top: ratios, along the SARS-CoV-2 genome, of site-specific A to G mutation counts on branches with > 90% SPRTA support vs. the same counts from all branches. A lower ratio means that more inferred A to G mutations at the considered site occur on phylogenetically uncertain branches. To avoid extreme ratios due to high variance at low counts, we only include sites with at least 50 substitutions of the considered type across all branches. Second, third and fourth rows: same for G to A, C to T, and T to C substitutions, respectively. Sites that have been most impacted by uncertainty are annotated with position and count information. Note that points are drawn with size inversely proportional to Y-axis values.

**Extended Data Fig. 7:**
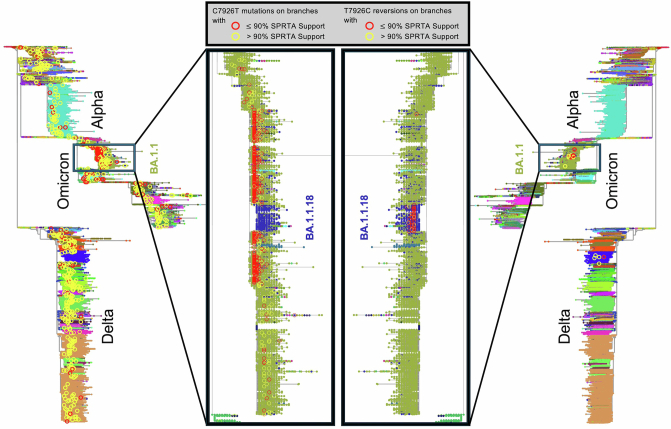
Complex mutational patterns at position 7926 of the SARS-CoV-2 genome. Far left: SARS-CoV-2 phylogeny as in Fig. 4, showing C to T substitutions at position 7926 as yellow circles (on branches with > 90% SPRTA support, *n* = 617) and red circles (on branches with < 90% SPRTA support, *n* = 716). Center left: Zoom-in to Omicron lineage BA.1.1, with *n* = 178 C to T substitutions on branches with > 90% SPRTA support, and *n* = 661 C to T substitutions on branches with < 90% SPRTA support. Far right and center right: The same phylogeny and zoom-in, now showing T to C reversions at the same position (full tree has 22 yellow circles and 43 red; zoom-in has 18 yellow circles and 42 red). The right-hand tree and zoom-in are in reverse orientation for ease of comparison. Low support substitutions (red circles) are prevalent in the zoom-in, showing elevated evolutionary uncertainty in this lineage and genome position.

**Extended Data Fig. 8:**
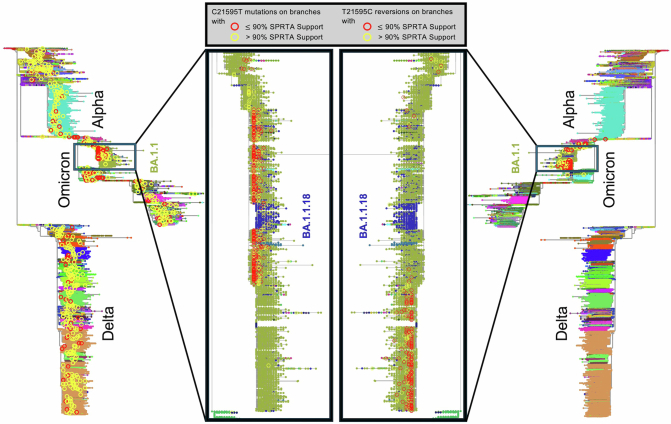
Complex mutational patterns at position 21595 of the SARS-CoV-2 genome. Far left and left: SARS-CoV-2 phylogeny as in Fig. 4 and zoom-in to Omicron lineage BA.1.1. Yellow and red circles are as in Fig. 7, this time showing C to T substitutions at position 21595 (874 yellow and 1129 red on the full tree; 429 yellow and 1024 red on the zoom-in). Far right and center right: The same phylogeny and zoom-in, now showing T to C reversions at the same position (full tree has 75 yellow circles and 180 red; zoom-in has 71 yellow circles and 175 red). Low support substitutions (red circles) are prevalent in the zoom-in, showing elevated evolutionary uncertainty in this lineage and genome position.

In phylogenetic investigations of mutation rates and selective pressures, mutations are usually estimated on a fixed phylogenetic tree, without accounting for phylogenetic uncertainty^22^. We find that ignoring phylogenetic uncertainty does not substantially affect the inference of genome-wide mean nucleotide substitution rates for SARS-CoV-2 (Extended Data Fig. 6a). However, it can substantially affect the numbers of inferred mutations at individual sites (Extended Data Fig. 6b). One of the sites strongly affected is 17040, owing to previously described uncertain reversions (Fig. 4). Other significantly affected sites also show similar patterns, with many reversions following a substitution (for example, see Extended Data Fig. 7 for position 7926 and Extended Data Fig. 8 for position 21595). The presence of several genome positions with high reversion rate and therefore highly uncertain mutational history might explain why we find lower SPRTA scores in real data than in simulations (Extended Data Fig. 1g). Details of these methods are provided in Methods, ‘Assessing the impact on mutation rates’.

### Effect on Pango lineages

The definition of pathogen outbreak lineages (for example, with the Pango^3^ or Autolin^26^ systems) and the assignment of samples to these lineages (for example, with the Pangolin tool^27^) often rely on a fixed phylogenetic tree. Uncertainty in this tree is typically ignored for these tasks. We investigated how much phylogenetic uncertainty might affect the definition of and assignment to Pango lineages, a globally important system for classifying and naming SARS-CoV-2 lineages including variants of concern (detailed methods in ‘Assessing the impact on Pango lineages’).

**Fig. 6:**
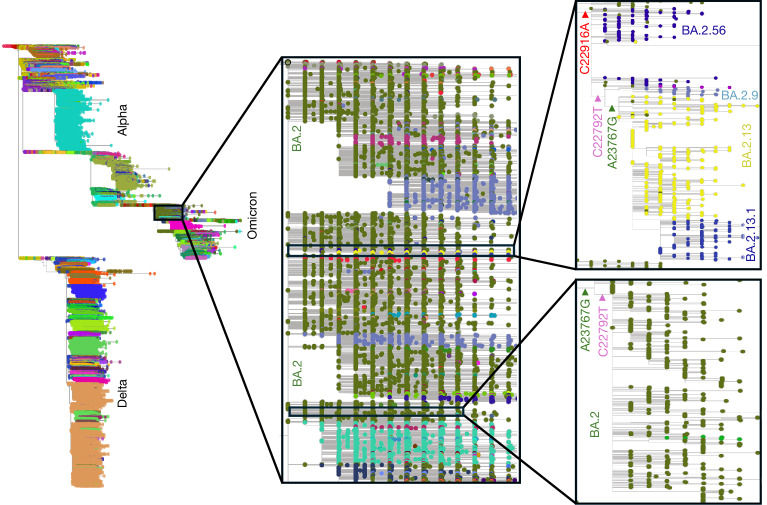
Uncertain origin of Pango lineage BA.2.13. Left, the global SARS-CoV-2 tree as in Fig. 4. Middle, magnified view of lineage BA.2. Right, magnified view of the two plausible origins of Pango lineage BA.2.13 within BA.2 and its descendants. Top right, the maximum-likelihood placement of BA.2.13 as a descendant of sub-lineage BA.2.56. BA.2.13 has three substitutions that are not part of BA.2: C22916A (red, shared with BA.2.56 and BA.2.9), C22792T (purple, shared with BA.2.9) and A23767G (green). The inferred maximum-likelihood tree suggests that BA.2.13 evolved through a A23767G mutation within a BA.2.56 background already containing substitutions C22916A and C22792T. Bottom right, the distinct occurrence of A23767G and C22792T within the BA.2 lineage. SPRTA shows that lineage BA.2.13 might plausibly have evolved from an additional C22916A substitution within this BA.2 background already containing substitutions A23767G and C22792T, corresponding to a reversed ordering of the three substitutions that define BA.2.13.

We found that out of 1,127 Pango lineages in our tree, 26 had more than 5% probability (and 39 had more than 1% probability) of origin from a different Pango lineage than the one implied by the maximum-likelihood tree. Among these, the most noticeable (due to its size and uncertainty) was Pango lineage BA.2.13. This lineage contains more than 500 genomes and is inferred to have descended from Pango lineage BA.2.56 in our maximum-likelihood tree (Fig. 6). However, a likely alternative placement of this lineage suggests that it might have evolved directly from BA.2. The uncertainty appears to be caused by the joint occurrence of the pair of substitutions A23767G and C22792T, both within lineages BA.2 and BA.2.56, giving rise to two almost equally probable evolutionary origins (with approximately 54% and 46% total SPRTA support, respectively) of BA.2.13 (Fig. 6).

**Extended Data Fig. 9:**
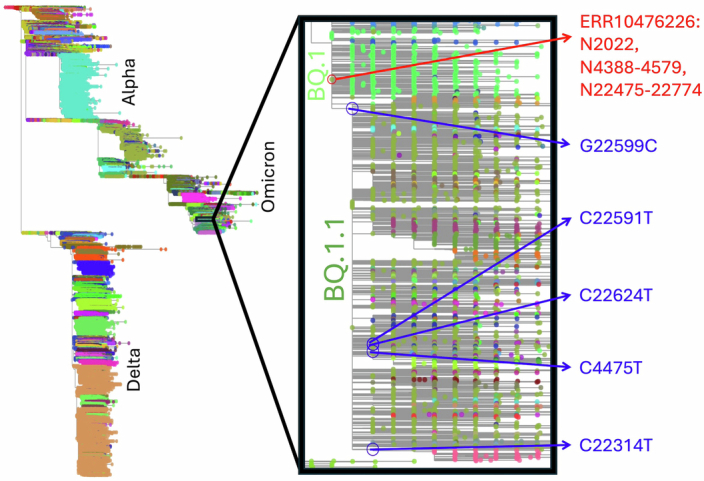
Uncertain placement and lineage assignment of sample ERR10476226. To left is the global SARS-CoV-2 tree as in Fig. 4. To right we zoom-in on the location in lineage BQ.1 of sample ERR10476226 (highlighted inside the red circle), and the five alternative placements of this sample (with blue circles, two of which overlap fully, highlighting the tree nodes descending from the branch of each alternative placement), all with the same likelihood and SPRTA score (≈ 16.7%) as the placement in the maximum likelihood tree. In red text after the sample name we list the regions of the consensus sequence that contain “N” characters (i.e. that are not informative). These positions overlap with the mutations (blue text) on branches of alternative placements of the sample. In particular, mutation G22599C separates BQ.1 and its descendant lineage BQ.1.1, and the “N” of the sample sequence at this position is the reason why it could be assigned to either lineage BQ.1 or BQ.1.1.

We also found that, out of the 2,072,111 genomes in our tree, 3,036 had less than 95% total SPRTA support for being assigned to the Pango lineage onto which they are placed in the maximum-likelihood tree; 754 of these had less than 70% support. We illustrate these findings using sample ERR10476226. This sample has 6 possible placements in the tree, all with the same likelihood and SPRTA support (approximately 16.7%). This is owing to the incomplete consensus sequence of the sample, causing it to be potentially identical to six different genomes in the tree (Extended Data Fig. 9), and so possibly being considered a ‘rogue taxon’. Although the maximum-likelihood placement in the tree of this sample is in Pango lineage BQ.1, all five other placements are on lineage BQ.1.1, which can therefore be interpreted as the most probable lineage assignment for this sample.

## Discussion

With the increasing use of genomic epidemiology, pandemic-scale phylogenetics is set to become an essential tool for pandemic preparedness and epidemiology in general. New approaches such as MAPLE^9^ and UShER^8^ can be used to infer the huge phylogenetic trees necessary at this scale, but there is considerable uncertainty inherent in these estimates due to complications including low divergence, recurrent mutations and errors, and incomplete genome sequences^21,28,29^.

Traditional methods to quantify and represent this uncertainty cannot be used with pandemic-scale datasets. To overcome this problem, we present a new approach, SPRTA. In addition to addressing the limitations of existing methods in terms of computational demand, SPRTA also offers a new mutational (or ‘lineage evolution’) interpretation of branch support scores that is particularly useful in genomic epidemiology, replacing the topological focus of previous approaches.

Owing to the excessive computational demand of applying Bayesian phylogenetic methods or Felsenstein’s bootstrap to large genome datasets, pandemic-scale phylogenies are usually taken at face value in downstream analyses such as inference of viral geographic spread^30^, mutational patterns^22^, lineage assignment^26^, recombination^31^ and variant fitness advantage^32^. Unchecked errors and uncertainty in phylogenetic trees can therefore propagate in these analyses and affect their accuracy and measures of uncertainty. Our approach allows us to efficiently distinguish between reliable and unreliable parts of an inferred phylogenetic tree, so that downstream analyses can focus on reliable phylogenetic signals or integrate over phylogenetic uncertainty.

Although SPRTA is currently implemented in MAPLE and optimized for applications to datasets with a low level of divergence, it would be possible to implement the same approach within any likelihood-based phylogenetic inference tool. Its mutationally focused scores could therefore be applied to any phylogenetic analysis, without constraints on the number of sequences considered, their divergence or their length. The only limitation to be considered is that at higher divergence, mutational histories conditional on a tree *T* become more uncertain, and so the mutation-based simulation benchmark that we used here might not be readily interpretable.

SPRTA could also be used to create a phylogenetic network based on a backbone maximum-likelihood phylogenetic tree that is extended to include additional branches with lower but substantial support. In this way, SPRTA could be used to efficiently summarize vast numbers of possible phylogenetic trees (see for example, ref. ^33^). In future, this might serve as a foundation for developing an efficient and complementary approach to Bayesian phylogenetics, helping to account for tree uncertainty in applications such as phylodynamics^34^.

In conclusion, SPRTA not only addresses a fundamental outstanding problem in genomic epidemiology, but also offers a new paradigm in evolutionary biology for the interpretation and representation of phylogenetic information and uncertainty.

## Methods

### SPRTA and aBayes

Given an estimated rooted phylogenetic tree *T* and data *D* in the form of a multiple sequence alignment, our aim is to assign confidence scores to branches *b* of *T*.

We take inspiration from the approximate Bayes (aBayes) approach^17^. aBayes assigns to *b* a probability score Pr(*b*∣*D*, *T*\*b*) based on the ratio of the likelihood Pr(*D*∣*T*) of the estimated binary tree *T* versus the likelihoods of the trees ${T}_{i}^{b}$ obtained using nearest neighbour interchange^36^ (NNI) moves centred around *b* (which comprise $T={T}_{1}^{b}$ itself in addition to two tree topologies not containing the clade in *T* descending from *b*):

2 $${\rm{aBayes}}(b)=\Pr (b| D,T\backslash b)=\frac{\Pr (D| T)}{{\sum }_{i=1}^{3}\Pr (D| {T}_{i}^{b})}.$$

These NNI moves perform small changes to *T* adjacent to branch *b*. One of the appeals of aBayes is that it can score not only *T*, but also the two alternative topologies considered, ${T}_{2}^{b}$ and ${T}_{3}^{b}$, not containing the clade defined by *b*:

3 $$\Pr ({T}_{j}^{b}| D,T\backslash b)=\frac{\Pr (D| {T}_{j}^{b})}{{\sum }_{i=1}^{3}\Pr (D| {T}_{i}^{b})}.$$

aBayes has a topological focus, with score aBayes(*b*) for branch *b* interpreted as the support for the existence of the clade of *T* containing all descendants of *b*. aBayes(*b*) is in effect an approximate Bayesian posterior score for this clade, where a flat tree prior is assumed. In contrast to typical Bayesian phylogenetics, however, instead of integrating over branch lengths we define $\Pr (D| {T}_{i}^{b})$ as the maximum-likelihood score of topology ${T}_{i}^{b}$ over all possible branch lengths, and we only consider the alternative topologies obtainable through a single NNI move^17^ starting from *T*. Although computationally much faster than Felsenstein’s bootstrap, aBayes can still be too demanding for large genomic epidemiological datasets if implemented within classical maximum-likelihood phylogenetic methods (for example, see Fig. 2). Also, aBayes is defined based only on NNI moves, an approach insufficiently comprehensive for pandemic-scale data^9^: owing to the existence of many phylogenetic topologies with similar likelihood^29^, the two alternative topologies obtainable through NNI moves, ${T}_{2}^{b}$ and ${T}_{3}^{b}$, might represent only a very small subset of plausible alternative topologies not containing the clade defined by *b*.

Here we address these limitations and define a new measure of branch support, SPRTA, that is particularly useful in the context of large-scale genomic epidemiology, but is also applicable more generally in phylogenetics. First, to address the problem of computational demand, we consider trees estimated using methods suitable for pandemic-scale datasets, such as MAPLE^9^. In the following, we assume that the likelihood of alternative tree topologies is also calculated with MAPLE or a similar method.

We define the SPRTA support of branch *b* by considering a certain number *I*_*b*_ of possible alternative topologies ${T}_{i}^{b}$ (1 ⩽ *i* ⩽ *I*_*b*_), obtained by performing single subtree prune and regraft^36^ (SPR) moves that relocate *S*_*b*_, the subtree of *T* containing all descendants of *b*, as a descendant of other parts of *T* (Fig. 1). Again, we assume for simplicity that ${T}_{1}^{b}=T$ corresponds to the null SPR move. Compared to NNI moves, SPR moves are much more numerous, and can cause long-range changes to the tree; as such, SPR moves create a much more comprehensive set of alternative evolutionary histories than NNI moves. For each branch *b*, the number of possible SPR moves is linear in the number of sequences in the considered dataset, which means if we do not select which of these SPR moves we focus on, *I*_*b*_ can become too large, and the calculation of SPRTA scores too computationally demanding. However, to obtain an accurate evaluation we only need to consider topologies that have non-negligible likelihood scores compared to *T*.

**Extended Data Fig. 10:**
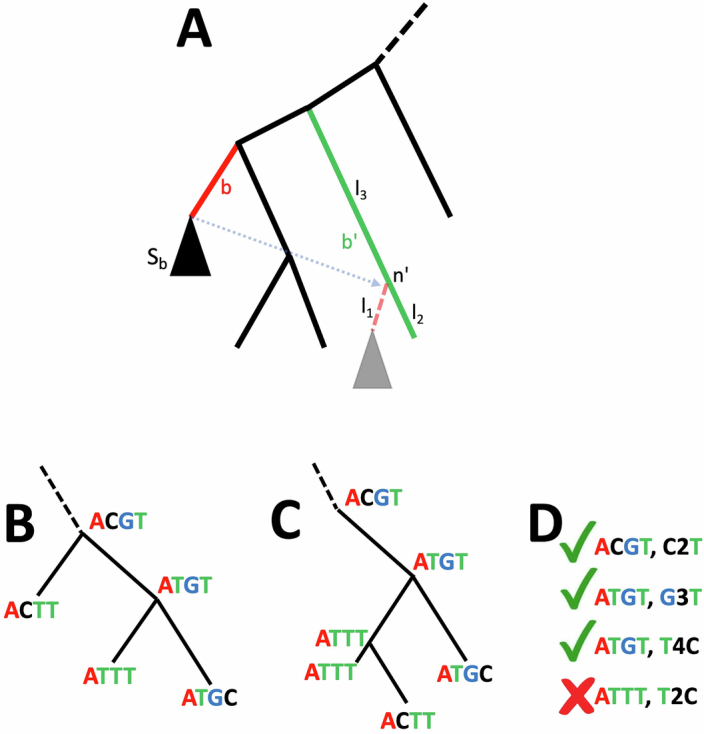
Graphical representation of branch length optimization and branch support assessment. **A** Given a branch *b* (highlighted in red), we show an example re-placement (dotted shaded blue arrow) of its subtree *S*_*b*_ (black triangle). ${n}^{^{\prime} }$ is the node at which *S*_*b*_ is re-attached, within branch ${b}^{^{\prime} }$ (green). To evaluate this SPR move, we optimize the branch lengths *l*_1_, *l*_2_ and *l*_3_ in the figure, while the lengths of the remaining branches in the tree are kept constant. Other details are as in Fig. 1. **B–D** To assess SPRTA in our simulation-based benchmark, we consider support values given to correctly and wrongly inferred mutational events. Here we give a graphical example. **B** Part of an example true, simulated tree (the dashed branch represents the remainder of the tree) annotated with simulated ancestral genome sequences. For simplicity, we consider a genome of length 4. **C** Example tree estimated from genomes in **B**, annotated with inferred ancestral genomes. **D** Mutation events inferred in **C**. The first three mutation events are also present in **B**, and so are classified as correctly inferred. The final mutation event, absent in **B**, is instead classified as wrongly inferred. We evaluate our branch support method based on the score given to branches with correctly inferred mutations (where higher scores are considered better) vs. branches with wrongly inferred mutations (lower scores considered better).

For this reason, we first perform an initial, approximate evaluation of alternative tree topologies obtained through SPR relocations of *S*_*b*_ using fixed branch lengths (we leave the length of the placement branch equal to the length of *b*, and we assess placements only halfway along branches). From this, we retain only topologies with an initial log-likelihood score difference from *T* within a threshold corresponding approximately to one extra mutation event (the natural logarithm of the genome length, which for SARS-CoV-2 is about 10.3). The likelihoods of all *I*_*b*_ topologies passing this threshold are then more deeply evaluated by optimizing branch lengths. This two-step approach is similar to the ‘baseball’ heuristic of the metagenomic query mapper pplacer^19^. More precisely, when we consider an alternative placement of *S*_*b*_ on branch ${b}^{^{\prime} }$, let us use ${n}^{^{\prime} }$ to denote the new node at the conjunction of ${b}^{^{\prime} }$ with *S*_*b*_. For this placement, the three branches whose length we optimize are the one connecting ${n}^{^{\prime} }$ with *S*_*b*_ (length *l*_1_), the one within ${b}^{^{\prime} }$ below ${n}^{^{\prime} }$ (length *l*_2_), and the one within ${b}^{^{\prime} }$ above ${n}^{^{\prime} }$ (length *l*_3_), similarly to the ‘lazy subtree rearrangement’ approach of RAxML^37^ (see Extended Data Fig. 10a). $\Pr (D| {T}_{i}^{b})$ is defined as the maximum likelihood obtained by optimizing these three branches, while leaving all other model parameters and branch lengths unaltered. This approximation of $\Pr (D| {T}_{i}^{b})$ is the same one made by MAPLE, and is justified by the fact that, at the low levels of divergence typically considered in genomic epidemiology, changes in topology and branch lengths usually only affect nodes near those directly affected by the changes^9^.

The likelihood score $\Pr (D| {T}_{i}^{b})$ of an SPR move is calculated by MAPLE (up to a normalizing factor) by comparing the partial likelihoods at node *B* (the root of *S*_*b*_), informed by sequence data within *S*_*b*_, against the partial likelihoods at the new placement nodes *A*_*i*_, informed by the sequence data within *T*\*S*_*b*_ (ref. ^9^). The partial likelihoods at node *B* represent genetic sequence uncertainty for the ancestor represented by this node. In genomic epidemiology, due to dense sampling and short divergence, this uncertainty is often negligible, and these partial likelihoods will often support only one genome. Likewise, partial likelihoods at *A*_*i*_ will often also only support a single genome. In these cases, the score $\Pr (D| {T}_{i}^{b})$ (up to a normalizing factor) is used as an approximation of the probability that the genome in *B* evolved from the genome in *A*_*i*_. In case uncertainty of the ancestral genetic sequence at these nodes is not negligible, we consider all possible ancestral genomes, weighted according to their likelihood, and $\Pr (D| {T}_{i}^{b})$ will approximate the probability that any of the possible genomes in *B* evolved from any of the possible genomes in *A*_*i*_.

Due to the initial filtering of plausible alternative topologies, *I*_*b*_ depends on branch *b*. These topologies are the same ones typically considered during the final stage of standard tree search in MAPLE.

SPRTA defines the support probability of *b* as

4 $${\rm{SPRTA}}(b)=\Pr (b| D,T\backslash b)=\frac{\Pr (D| T)}{\sum _{1\leqslant i\leqslant {I}_{b}}\Pr (D| {T}_{i}^{b})}$$

where *T* \ *b* represents the two subtrees obtained by removing *b* from *T*. We can similarly define $\Pr ({T}_{i}^{b}| D,T\backslash b)$ for the alternative placements ${T}_{i}^{b}\ne T$ (2 ⩽ *i* ⩽ *I*_*b*_) for any subtree *S*_*b*_ considered. Note that SPRTA typically evaluates a much larger number of alternative topologies than aBayes (equation (2)): up to quadratic in the number of genomes analysed for SPRTA, but only linear for other local support measures such as aBayes or aLRT.

When evaluating possible SPR placements, placements resulting in the same topology are equivalent, and so we only consider them once. In particular, we take into account multifurcations caused by branches of length 0, and consider placements into points of the tree with a null distance between them (based on considering optimized placement-specific branch lengths as just described) as equivalent.

Because relative likelihood calculations of alternative topologies are performed as a standard component of tree inference in MAPLE, assessing SPRTA support probabilities adds negligible computational overhead.

MAPLE infers rooted phylogenetic trees, and as such we have defined and implemented SPRTA to assess rooted tree inference. The same principles could however also be applied to the evaluation of unrooted trees. In this case, to calculate the score of a branch *b*, we would not only consider SPR moves representing alternative placements of subtree *S*_*b*_ within *T* \ *S*_*b*_, but also SPR moves representing alternative placements of *T* \ *S*_*b*_ within *S*_*b*_, so increasing the number of SPR moves to be considered for each branch *b*, but leaving the rest of the approach unaltered.

### Benchmarking of SPRTA support

Branch support measures are often used to assess the expected accuracy of phylogenetic inference. However, how do we assess the accuracy of these measures of accuracy? We approach this problem using simulations, for which we have a ground truth against which to compare estimates. First, we simulate a tree and a set of genomes (Extended Data Fig. 10b); second, we estimate a tree from these simulated genomes (Extended Data Fig. 10c). Some branches of the inferred tree will be correct and some will be wrong: therefore, third, we assess branch support measures according to their ability to give higher support scores to correctly estimated branches, and lower support scores to wrongly estimated branches (Extended Data Fig. 10d).

We benchmark branch support methods on simulated SARS-CoV-2 genome data (Methods, 'Simulated genomes’) using our ‘mutational’ focus rather than a traditional ‘topological’ focus. We define phylogenetic correctness in terms of the genome evolutionary history implied by a phylogenetic tree. From each individual simulated dataset (see graphical example in Extended Data Fig. 10b), we first infer in MAPLE a maximum-likelihood phylogenetic tree *T* and mutation events by marginal posterior mutation mapping^38^ conditional on *T* (Extended Data Fig. 10c). Only mutation events inferred with  >0.5 probability by MAPLE are considered here. We define estimated mutation events as pairs (*G*, *m*) where *G* is a whole genome sequence and *m* = (*n*_1_, *p*, *n*_2_) is a single-nucleotide substitution at position *p* of *G* from nucleotide *n*_1_ (contained in *G* at position *p*) to nucleotide *n*_2_ (Extended Data Fig. 10d). An inferred mutation event (*G*, *m*) is considered correct if it is also present in the simulated tree, otherwise it is considered as an estimation error (Extended Data Fig. 10d). Finally, we assign the support score of branch *b* (estimated by SPRTA or any other method) to all the mutations inferred to have occurred on *b*. We consider a branch support measure as more accurate if in our simulations it assigns higher support to correctly estimated mutation events, and lower support to erroneously inferred ones.

Evaluating the accuracy of SPRTA using inferred mutations might seem counter-intuitive, since our definition of SPRTA scores does not consider explicit mutational histories. However, by analysing the levels of support for different placements of subtree *S*_*b*_ associated with branch *b*, different possible mutation histories immediately ancestral to *S*_*b*_ are implicitly considered and evaluated via the likelihood of alternative subtree placements. We thus interpret SPRTA scores as the support for the hypothesis that the profile *B* at the lower end of *b* evolved from profile *A* at the upper end of *b* through mutations on branch *b* (see ‘Accuracy’).

In the scenario of short branches considered here, there is extremely low uncertainty in most mutation events and ancestral genomes implied by a given topology, and so we can often interpret the correctness of the mutational history inferred on *b* as the correctness of *b* itself. This does not mean that in this case *b* will be topologically correct—for example, the placement of rogue taxa within or outside the subtree *S*_*b*_ defined by *b* can make *b* topologically uncertain without causing uncertainty in the inferred mutational history or the placement of *S*_*b*_.

### Implementation and usage of SPRTA

We ran SPRTA as implemented within MAPLE v.0.6.8 (https://github.com/NicolaDM/MAPLE). While SPRTA can be run in MAPLE at the same time as tree inference with minimal additional computational cost (Methods ‘SPRTA and aBayes’), to aid comparability of computational performance with other approaches here we have considered its use to assess a pre-estimated input phylogenetic tree provided with the option –inputTree. We used options –numTopologyImprovements 0 –doNotImproveTopology to perform a shallow SPR search in MAPLE. We also used options –model UNREST –rateVariation to use an UNREST model^39^ with rate variation^21^, and option –estimateMAT to infer mutation events.

### Other branch support methods

All other branch support measures considered here were calculated using IQ-TREE v.2.1.3^40^ with options –seqtype DNA –seed 1 -m GTR+F+G4 –quiet -nt 1. As with SPRTA, we always use the tree estimated by MAPLE as a starting tree (supplied via the option -t) since on these datasets IQ-TREE will typically not converge to a tree with likelihood as high as MAPLE^9^. We used the following additional IQ-TREE options:

-B 1000 (1,000 bootstrap replicates) for UFBoot2^7^
–fast -b 100 (100 bootstrap replicates and fast tree search) for Felsenstein’s bootstrap^2^
–fast -b 100 –tbe (100 bootstrap replicates and fast tree search) for TBE^10^
–fast –alrt 1000 (1,000 bootstrap replicates and fast tree search) for aLRT-SH^16^
–fast –alrt 0 (fast tree search) for aLRT^15^
–fast –abayes (fast tree search) for aBayes^17^
–fast –lbp 1000 (1,000 bootstrap replicates and fast tree search) for LBP^14^.

The number of bootstrap replicates has very limited impact on the computational demand of UFBoot2 and LBP, hence these were set to 1,000 for reduced stochasticity with minimal computational cost.

### SARS-CoV-2 genome datasets

#### Viridian genome dataset

We applied SPRTA to a SARS-CoV-2 dataset containing 2,072,111 genomes collected up to February 2023. The consensus sequences of these genomes were consistently called with Viridian, a tool that prevents common reference biases in genomic regions of low sequencing coverage^23^. Furthermore, we filtered out potentially contaminated samples, and masked alignment columns affected by recurrent sequence errors^21^. We estimated a phylogenetic tree using MAPLE v.0.6.8 with an UNREST substitution model, rate variation, and deep SPR phylogenetic search. For a full description of data preparation and phylogenetic inference, see ref. ^21^. We ran SPRTA on this alignment and tree using MAPLE v.0.6.9, with the options described in ‘Implementation and usage of SPRTA’, and additionally with option –supportFor0Branches to evaluate the support of all genome placements, even those not involving mutations (see ‘Uncertainty of SARS-CoV-2 evolution’).

#### Simulated genomes

For benchmarking, we simulated SARS-CoV-2 genomes evolving along a known (‘true’) background phylogeny. The background tree we used was the publicly available 26 October 2021 global SARS-CoV-2 phylogenetic tree from http://hgdownload.soe.ucsc.edu/goldenPath/wuhCor1/UShER_SARS-CoV-2/^41^ representing the evolutionary relationship of 2,250,054 SARS-CoV-2 genomes, as inferred using UShER^8^.

We used phastSim v.0.0.3^42^ with options –treeFile public-latest.all.nwk –scale 0.00003344 –reference MN908947.3.fasta –alpha 0.2 –createNewick to simulate sequence evolution along this tree according to SARS-CoV-2 non-stationary neutral mutation rates^22^, using the SARS-CoV-2 Wuhan-Hu-1 genome^43^ as root sequence, and with gamma-distributed (*α* = 0.2) rate variation^44^ (similar to that estimated from real data^21^). These simulations of complete genomes were used for Figs. 2 and 3, and Extended Data Figs. 1e, 2 and 3.

We also created a second set of simulations mimicking the distribution of genome incompleteness from real data as in ref. ^9^. In each simulated sequence we included N and gap (–) characters copied in number and location from a randomly selected paired sequence from the real SARS-CoV-2 genome dataset considered in ref. ^9^. This step simulates the distribution of missing sequence data due to low sequencing depth at certain specific genome regions. Additionally, in each simulated sequence we masked a number of randomly selected SNPs (differences with respect to the reference genome) equal in number to the isolated ambiguous characters in the paired randomly sampled real sequence. This additional step mimics the pattern caused by mixed infections and contamination, in which phylogenetically informative positions are selectively masked in consensus genomes due to within-sample heterozygosity (see ref. ^9^ for more detail). This second set of simulations was used to create Extended Data Figs. 1a–d,f, 4 and 5.

### Assessing the impact on mutation rates

To assess the impact of phylogenetic uncertainty on estimates of mutation patterns, we mimicked typical studies of mutation rate inference in SARS-CoV-2^22,45,46^. We consider real SARS-CoV-2 data and the corresponding inferred tree and mutation events as described in ‘Viridian genome dataset’. We then created three datasets: one containing all inferred mutations, one containing only mutations on branches with at least 50% SPRTA support (representing a mildly conservative approach, discarding highly uncertain mutations), and one with only mutations on branches with at least 90% SPRTA support (representing a highly conservative approach, removing any moderately uncertain mutation).

Equilibrium frequencies (Extended Data Fig. 6a) represent the equilibrium nucleotide distribution of the Markov chain defined by the genome-wide mutation rate matrix^47^ inferred from the mutation counts as

5 $${q}_{ij}\propto \frac{{n}_{ij}}{{{\rm{\pi }}}_{i}},$$

where *n*_*ij*_ is the count of mutations from nucleotide *i* to nucleotide *j* and π_*i*_ is the frequency of nucleotide *i* in the reference genome.

We assess the impact of phylogenetic uncertainty on site-specific mutation counts by calculating, for every genome position, the ratio of the most conservative mutation counts (those on branches with at least 90% support) to the least conservative mutation counts (those on all branches) (Extended Data Fig. 6b). To avoid high variance in values of this ratio at sites with low numbers of substitutions, only sites with at least 50 mutations of the given type over all branches were included.

### Assessing the impact on Pango lineages

To assess the impact of phylogenetic uncertainty on the definition and the inference of the origin of Pango lineages, we mapped 1,542 Pango lineage consensus genomes (as of 28 February 2023; https://github.com/corneliusroemer/pango-sequences) onto our SARS-CoV-2 phylogenetic tree (‘Viridian genome dataset’) using MAPLE v.0.7.3. This mapping associates Pango lineages with nodes in our tree. Similarly to our SARS-CoV-2 alignment, we masked regions of recurrent sequence errors from these consensus genomes (see ref. ^21^). Of these genomes, 1,127 mapped onto our tree with ≤1 mutation separating them from the tree, and with ≥95% SPRTA placement support score. We discarded consensus genomes that were further removed (>1 mutation separating them from the tree), as they typically represent more-recent lineages that did not exist at the time our dataset was collected. Consensus genomes with low SPRTA placement support were discarded since they could not be uniquely associated with a single node in our tree (this uncertainty can be caused, for example, by incomplete genome sequences in our dataset). In total, these 1,127 genomes mapped onto 1,117 distinct tree branches, and represent the Pango lineages that we can place on the tree with high confidence.

We used these 1,127 consensus genome placements to assign a Pango lineage to each branch and sample in our tree: each was assigned to the lineage represented by the closest ancestral consensus genome placement (the first one met moving from the considered node towards the tree root). We used this assignment of Pango lineages to assess uncertainty in the lineage assignment of the genomes in our dataset. For each of the 2,072,111 genomes in our alignment, we considered the SPRTA scores of their current and alternative placements. To each considered placement, we assigned the Pango lineage of the placement branch (alternative placements on the same branch as the placement of a Pango consensus genome were ignored).

### Reporting summary

Further information on research design is available in the Nature Portfolio Reporting Summary linked to this article.

## Online content

Any methods, additional references, Nature Portfolio reporting summaries, source data, extended data, supplementary information, acknowledgements, peer review information; details of author contributions and competing interests; and statements of data and code availability are available at 10.1038/s41586-025-09567-x.

## Supplementary information

Reporting Summary

## Data Availability

The real data alignment, metadata, inferred tree and SPRTA support scores are available on Zenodo (10.5281/zenodo.14974813)^24^. The tree used as input in our simulations was downloaded from http://hgdownload.soe.ucsc.edu/goldenPath/wuhCor1/UShER_SARS-CoV-2/. Pango lineage consensus sequences were downloaded from https://github.com/corneliusroemer/pango-sequences.

## Code availability

We ran SPRTA as implemented within MAPLE v.0.6.8, v.0.6.9 and v.0.7.3 (https://github.com/NicolaDM/MAPLE), but we also provide an implementation within CMAPLE^48^ which is incorporated into IQ-TREE v.3^49^. All other branch support methods considered were run as implemented within IQ-TREE v.2.1.3^40^. Code for the simulation-based method benchmark analysis is available at https://github.com/NicolaDM/MAPLE/blob/main/scripts/benchmarking_branchSupport.py.
